# Editorial Correspondence

**Published:** 1877-12

**Authors:** R. W. Westmoreland

**Affiliations:** Fulton, Arkansas


### EDITORIAL CORRESPONDENCE.

FULTON, ARKANSAS, December 7, 1877.

*Editor Atlanta Medical and Surgical Journal:*

I have just examined a case, which, from its interesting, and, to me, peculiar character, I deem worthy of publication. To many it will, no doubt, prove interesting as to me, and may, too, be in a manner profitable. The disease being located in the tongue—an organ whose functions serve so many important purposes, varied, however, in different individuals, and the loss of which occasions so much inconvenience—it follows that he who can successfully deal with this, and *all* other affections to which it is subject, is entitled to gratitude and reward.

The subject, a boy six years old, in every other respect perfectly healthy, has an erectile tumor of the tongue, of a nævus character. Various physicians have been consulted, and various plans of treatment adopted, such as ointments, bandages, etc., and all with the same unsuccessful result. The tumor is gradually growing larger, and is occasionally temporarily enlarged by local or constitutional disturbance, such as sudden change of temperature, irritants, etc. The general deformity about the mouth and throat, resulting from it, is very great. Besides the protrusion, which is considerable, the teeth and maxilla are decidedly abnormal, presenting a spectacle truly disgusting, were it not for the sympathy one is bound to feel for the little unfortunate. He may be truly said to have over a "mouthful" of the tumefied tongue, yet, strange to say, articulation is pretty fair, and mastication almost pefect. In connection with the general malformation of the tongue and mouth, abscesses of the submaxillary and sublingual glands exist. The throat just beneath the maxillary bones is very much swollen from the glandular disease, and resembles that of a very fat hog. The abscesses were once opened, and discharged a large quantity of pus, and at present fluctuation is very distinct.

After this rather imperfect description of the case, I proceed to give my opinion of its pathological character, mode of treatment, etc. I also desire your opinion on the subject. According to Erichsen, nævus of the tongue is rarely met with. He mentions a case similar to the one under consideration, as peculiar and novel. This is evidently of the variety of nævus called venous—its dark purple color being unlike artereal anastimosis, and its locality, as well as appearance, precluding the idea of its capillary nature. I conclude that the maxillary swelling depends on the enlarged protruding tongue producing tension and irritation of these parts resulting in inflammation and suppuration. In the treatment of venous nævus, four plans may be considered: 1st. To excite adhesive inflammation by seatons or stimulating injections. 2d. Excision by the knife. 3d. Removal by ligature. 4th. Removal by escharotics. On account of the danger of serious hemorrhage, I am inclined to favor the ligature, or that which acts on the same principle and is preferable—the ecraseur. The locality and size of the tumor render this plan more desirable. The line of demarkation between the redundant and normal tissue is very distinct.

I hope, but with misgivings, I confess, that success may crown my effort at relief, but will, at some future time, report the result of whatever treatment may be adopted.

R. W. Westmoreland, M.D.

---

